# The Coumarin Psoralidin Enhances Anticancer Effect of Tumor Necrosis Factor-Related Apoptosis-Inducing Ligand (TRAIL)

**DOI:** 10.3390/molecules17066449

**Published:** 2012-05-29

**Authors:** Joanna Bronikowska, Ewelina Szliszka, Dagmara Jaworska, Zenon P. Czuba, Wojciech Krol

**Affiliations:** Chair and Department of Microbiology and Immunology, Medical University of Silesia in Katowice, Jordana 19, 41-808 Zabrze, Poland; Email: jbronikowska@sum.edu.pl (J.B.);eszliszka@sum.edu.pl (E.S.); djaworska@sum.edu.pl (D.J.); zczuba@sum.edu.pl (Z.P.C.)

**Keywords:** coumarin, psoralidin, apoptosis, TRAIL, cancer cells, chemoprevention

## Abstract

Coumarins are a very common type of secondary plant metabolites with a broad spectrum of biological activities. Psoralidin is a naturally occurring furanocoumarin isolated from *Psoralea corylifolia* possessing anticancer and chemopreventive properties. Tumor necrosis factor-related apoptosis-inducing ligand (TRAIL) triggers apoptosis in cancer cells with no toxicity toward normal tissues. Endogenous TRAIL plays an important role in immune surveillance and defence against cancer cells. Coumarins can modulate TRAIL-mediated apoptosis in cancer cells. We examined the cytotoxic and apoptotic activities of psoralidin in combination with TRAIL on HeLa cancer cells. The cytotoxicity was measured by MTT and LDH assays. The apoptosis was detected using annexin V-FITC staining and mitochondrial membrane potential was evaluated using DePsipher staining by fluorescence microscopy. Death receptor (TRAIL-R1/DR4 and TRAIL-R2/DR5) expression was analyzed using flow cytometry. Psoralidin enhanced TRAIL-induced apoptosis in HeLa cells through increased expression of TRAIL-R2 death receptor and depolarization of mitochondrial membrane potential. Our study indicated that psoralidin augmented the anticancer effects of TRAIL and confirmed a potential use of coumarins in cancer chemoprevention.

## 1. Introduction

Bioactive compounds from fruits, vegetables, spices and herbs are widely considered to be valuable for human health [1,2,3]. Coumarins (1,2-benzopyrones) constitute an important and large class of oxygen heterocycles, often found as secondary plant metabolites. They are categorized and subdivided as follows: simple coumarins, furanocoumarins (linear and angular types), pyranocoumarins (linear and angular types), dicoumarins, and phenylcoumarins. Coumarins naturally present in many plants (primarily in angiosperms) possess anticoagulant, antimicrobial, antioxidant, anti-inflammatory, anti-allergic and anticancer properties [4,5].

**Figure 1:**
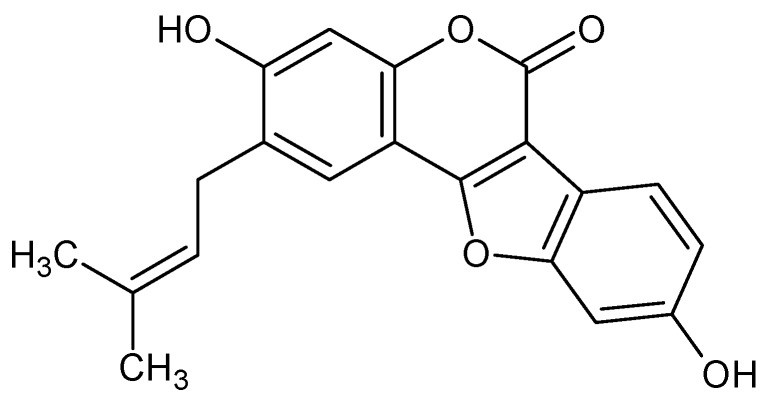
Chemical structure of psoralidin.

Psoralidin (Figure 1) is one of the major (angular type) furanocoumarins isolated from the seeds of *Psoralea corylifolia* (Leguminosae), a medicinal plant widely distributed in Southeastern Asian countries [6,7]. Psoralidin has been shown to induce cytotoxicity against gastric (SNU-1, SNU-16), colon (HT-29) and breast (MCF-7) cancer cells. Psoralidin also caused apoptosis in androgen-dependent (LNCaP, C4-2B) and androgen-independent (DU-145, PC-3) prostate cancer cells and inhibited growth of PC-3 xenograft tumor in nude mice [8,9,10,11,12].

Chemoprevention is a method of tumor control in which malignancy is prevented or reversed by nutritional or pharmacological intervention using natural or synthetic substances [13,14,15,16,17,18]. The role of bioactive compounds in cancer prevention has been confirmed in numerous laboratory and epidemiological studies [18,19,20,21,22]. Coumarins or their synthetic derivatives have been found to exhibit a variety of biological activities and have raised considerable interest because of their potential beneficial effects of human health and chemoprevention [4,5]. Therefore, the development of natural or synthetic coumarins is becoming increasingly recognized as an effective strategy in cancer prevention.

Tumor necrosis factor-related apoptosis-inducing ligand (TRAIL), a potent stimulator of apoptosis in cancer cells, is an important immune effector molecule in the surveillance and defence against developing tumors. Endogenous TRAIL is expressed on the surface of T lymphocytes, natural killer cells, dendritic cells, neutrophils, monocytes or macrophages and can be cleaved into a soluble, secreted form [18,23,24]. TRAIL triggers apoptosis in cancer cells through its interaction with specific death receptors. There are two receptors, TRAIL-R1/DR4 and TRAIL-R2/DR5, that by extracellular domains recognize and bind ligand. The death receptors contain complete and functional intracellular death domains responsible for the activation of apoptosis pathway in cancer cells [25,26]. However, some cancer cells are resistant to TRAIL-mediated death. The expression of the death receptors in cancer cells could be involved in TRAIL-resistance [23,24,25,26].

Our previous findings demonstrated that psoralidin augmented TRAIL-mediated apoptosis and overcame TRAIL-resistance in LNCaP prostate cancer cells [27]. In the present study we report for the first time the molecular mechanism by which psoralidin enhances TRAIL-induced apoptosis in cancer cells on HeLa cell line model. The TRAIL-induced apoptotic signaling pathway is a potential target for the coumarins in the tumor cells. Overcoming of TRAIL-resistance by psoralidin may be one of the mechanisms responsible for the cancer chemopreventive properties of coumarins. 

## 2. Results and Discussion

### 2.1. Cytotoxic and Apoptotic Activities of Psoralidin in HeLa Cancer Cells

**Figure 2:**
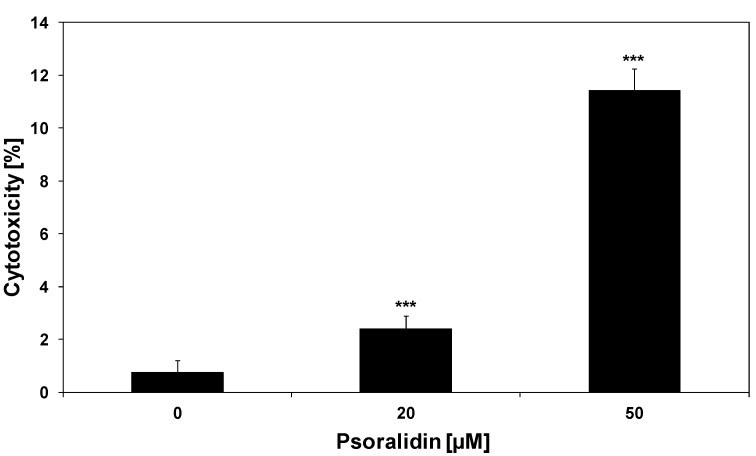
Cytotoxic activity of psoralidin in HeLa cancer cells. The cancer cells were incubated for 48 h with psoralidin at the concentrations of 20 μM and 50 μM. The percentage of cell death was measured by MTT cytotoxicity assay. The values represent mean ± SD of three independent experiments performed in quadruplicate (n = 3) (***p < 0.001 compared with control).

Psoralidin is one of the most active ingredients identified in *P. corylifolia*. The extracts from seeds of *P. corylifolia*, as well as psoralidin, possess antimicrobial, antioxidant, anti-inflammatory, antimutagenic and anticancer activities [6,7,27,28,29,30,31,32,33]. Previous experimental data from *in vitro* and *in vivo* studies showed that psoralidin induced cytotoxicity and apoptosis in cancer cells [9,10,11,12,27]. We tested cytotoxic and apoptotic activities of psoralidin against HeLa cells after 48 incubation time. Psoralidin at concentrations of 20–50 μM induced 2.4 ± 0.5%–11.4 ± 0.8% cytotoxicity in HeLa cells in a concentration-dependent manner (Figure 2). Our results indicate that cytotoxic effect of the compound against HeLa cells was mediated through apoptosis. The percentage of necrotic cells examined by lactate dehydrogenase assay and fluorescence microscopy with Ethidium Homodimer III was near 0%. At the concentration of 50 μM psoralidin induced 13.5 ± 1.2% apoptosis in HeLa cells. 

### 2.2. Cytotoxic and Apoptotic Activities of TRAIL in HeLa Cancer Cells

**Figure 3:**
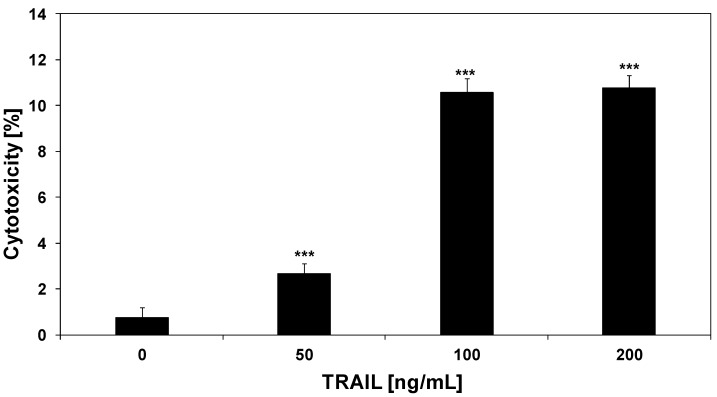
Cytotoxic activity of TRAIL in HeLa cancer cells. The cancer cells were incubated for 48 h with TRAIL at the concentrations of 50–200 ng/mL. The percentage of cell death was measured by MTT cytotoxicity assay. The values represent mean ± SD of three independent experiments performed in quadruplicate (n = 3) (***p < 0.001 compared with control).

TRAIL is a member of the tumor necrosis factor (TNF) superfamily, which includes potent inducers of apoptosis in a wide variety of cancer cells [23,24]. Recombinant human TRAIL used in our study is a soluble protein based on a natural endogenous ligand. We and others have reported that HeLa cells are resistant to TRAIL-mediated death [25,34,35,36]. TRAIL induced 2.7 ± 0.4%–10.8 ± 0.7% cytotoxicity in HeLa cells in a concentration-dependent manner (Figure 3). TRAIL induced the cytotoxic effect in cancer cells via the apoptotic route. The necrotic cell death percentage of HeLa cells examined by lactate dehydrogenase assay and fluorescence microscopy with Ethidium Homodimer III was near 0%. The apoptotic effect of TRAIL at a concentration of 100 ng/mL was 10.8 ± 1.0%. Concentrations of TRAIL of 200 ng/mL or higher did not significantly increase the cytotoxic and apoptotic effects on HeLa cells (our unpublished observations).

### 2.3. Cytotoxic and Apoptotic Activities of TRAIL in Combination with Psoralidin in HeLa Cancer Cells

Several cellular mechanisms contribute to the overall anti-cancer activity of coumarins. TRAIL is considered to be a tumor-selective, apoptosis-inducing cytokine and TRAIL-mediated apoptotic pathway is a potential target for bioactive coumarins. TRAIL-resistant cancer cells can be sensitized to TRAIL-mediated cytotoxicity by various compounds [25,34,35,36,37,38,39,40]. 

**Figure 4:**
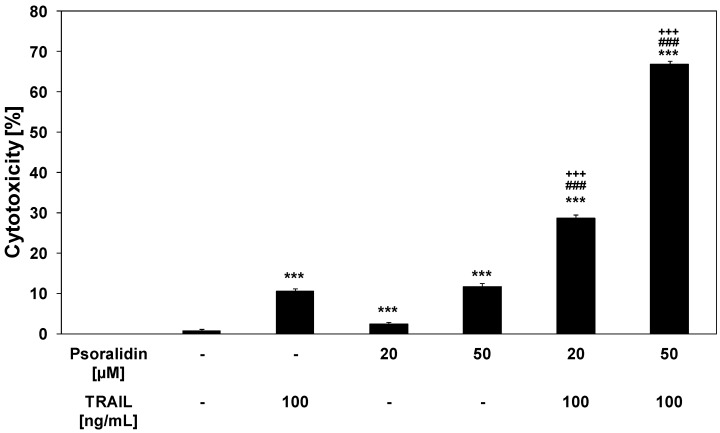
Cytotoxic activity of TRAIL in combination with psoralidin in HeLa cancer cells. The cancer cells were incubated for 48 h with TRAIL at the concentration of 100 ng/mL and psoralidin at the concentrations of 20 μM and 50 μM. The percentage of cell deaths was measured by MTT cytotoxicity assay. The values represent mean ± SD of three independent experiments performed in quadruplicate (n = 3) (***p < 0.001 compared with control, ^###^*p* < 0.001 compared with psoralidin and ^+++^*p* < 0.001 compared with TRAIL).

We investigated the cytotoxic and apoptotic activity of TRAIL in combination with psoralidin on HeLa cancer cells. The cytotoxicity of TRAIL at the concentration of 100 ng/mL in combination with psoralidin at the concentrations of 20 μM and 50 μM in HeLa cells is demonstrated in Figure 4. As shown in Figure 2 and Figure 3, little cytotoxicity was observed with psoralidin or TRAIL alone. HeLa cell cotreatment with TRAIL and psoralidin increased the percentage of cell deaths to 28.7 ± 0.8%–66.9 ± 0.8%. Using Webb’s method [41] it was calculated that for TRAIL at the concentration of 100 ng/mL in combination with psoralidin at the concentrations of 20 μM and 50 μM, the synergistic effects were 13% and 21%, respectively. The obtained experimental values are higher than calculated and confirm this synergistic effect.

**Figure 5:**
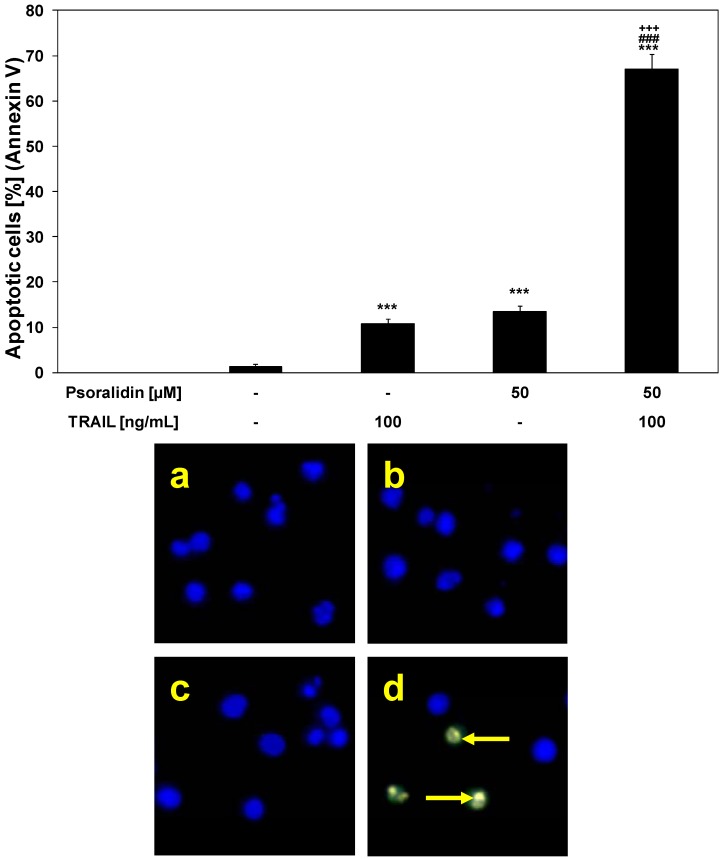
TRAIL induced apoptosis in combination with psoralidin in HeLa cancer cells. The cancer cells were incubated for 48 h with TRAIL at the concentrations of 100 ng/mL and psoralidin at the concentration of 50 μM. Detection of apoptotic cell deaths by fluorescence microscopic using annexin V-FITC, EthidiumHomodimer III and Hoechst 33342 staining: (**a**) control cells, (**b**) cells incubated with TRAIL, (**c**) cells incubated with psoralidin, (**d**) cells incubated with TRAIL and psoralidin. The values represent mean ± SD of three independent experiments performed in quadruplicate (n = 3) (***p < 0.001 compared with control, ^###^*p* < 0.001 compared with psoralidin and ^+++^*p* < 0.001 compared with TRAIL). The healthy cells (stained with Hoechst 33342) emitted blue fluorescence and apoptotic cells (stained with Annexin V-FITC and Hoechst 33342) emitted green and blue fluorescence. Cells undergoing apoptosis showed nuclei shrinkage, chromatin condensation and nuclei fragmentation, indicated by arrows.

Psoralidin in combination with TRAIL induced cytotoxic effects in cancer cells via the apoptotic route. The necrotic cell death percentage of HeLa cells examined by lactate dehydrogenase assay and fluorescence microscopy with Ethidium Homodimer III was near 0%. Psoralidin significantly enhanced TRAIL-induced apoptosis in HeLa cancer cells (Figure 5). 

The percentage of apoptotic cells stained with annexin V-FITC detected by fluorescence microscopy after a 48-hour exposure to TRAIL at a concentration of 100 ng/mL and psoralidin at a concentration of 50 μM was elevated to 67.1 ± 3.3%. Like in the case of the cytotoxicity the calculated synergistic effect was 23%. The obtained experimental values are higher than calculated and confirm this synergistic effect.

TRAIL plays a significant role in immune surveillance and defense mechanisms against tumor cells [18,23]. The results suggest that psoralidin can sensitize cancer cells to immune effector mechanisms such as TRAIL-mediated apoptosis.

Our previous findings demonstrated that psoralidin reversed TRAIL-resistance and significantly augmented the apoptotic activity of TRAIL in LNCaP prostate cancer cells [27]. Srinivasan *et al.* reported the induction of apoptosis by psoralidin in DU-145 and PC-3 prostate cancer cells. The cancer cells were co-treated with psoralidin and recombinant TNF-alpha, that belongs also like TRAIL to the TNF superfamily. The authors confirmed that psoralidin overcame TNF-alpha-mediated resistance in prostate cancer cell lines [11].

Other coumarins also sensitize cancer cells to TRAIL-mediated death. Esculetin (6,7-dihydroxy-coumarin) is a coumarin with chemopreventive and anticancer properties found in *Artemisia capillaries*, *Citrus limonia* and *Euphorbia lathyris*. Kok *et al.* demonstrated the enhancement of TRAIL-induced apoptosis in SAS oral cancer cells by esculetin [42]. 

There are many factors contributing to the resistance to TRAIL-induced apoptosis [18,23]. Therefore, further investigations will be required to explain the molecular mechanisms by which psoralidin sensitize cancer cells to TRAIL-mediated death.

### 2.4. The Mechanism by Which Psoralidin Sensitize HeLa Cancer Cells to TRAIL-Induced Apoptosis

Coumarin and its natural or synthetic derivatives exert anticancer potential against HeLa cells. Numerous findings confirmed that coumarins induced programmed death in cancer cells targeting several molecules associated with multiple signaling pathways [43,44,45,46].

#### 2.4.1. Effects of Psoralidin on Death Receptor Expression in HeLa Cancer Cells

TRAIL triggers cell death in various cancers through its interaction with death receptor TRAIL-R1/DR4 and/or TRAIL-R2/DR5, which contain a cytoplasmic death-domains capable of recruiting apoptosis signaling molecules and inducing apoptosis [18,23,25]. However, many tumor cells are resistant to TRAIL-mediated apoptosis [16,17,18,24,25,26,27,34,35,36,37,38,39,40,27,34]. Various natural agents have been demonstrated to augment TRAIL-induced apoptosis through induction of TRAIL-R2 expression, indicating that death receptor expression levels might be involved in resistance to TRAIL [16,18,25,26,47]. Thus, induction of TRAIL-R2 expression could enhance cytotoxicity and apoptosis mediated by TRAIL.

**Figure 6:**
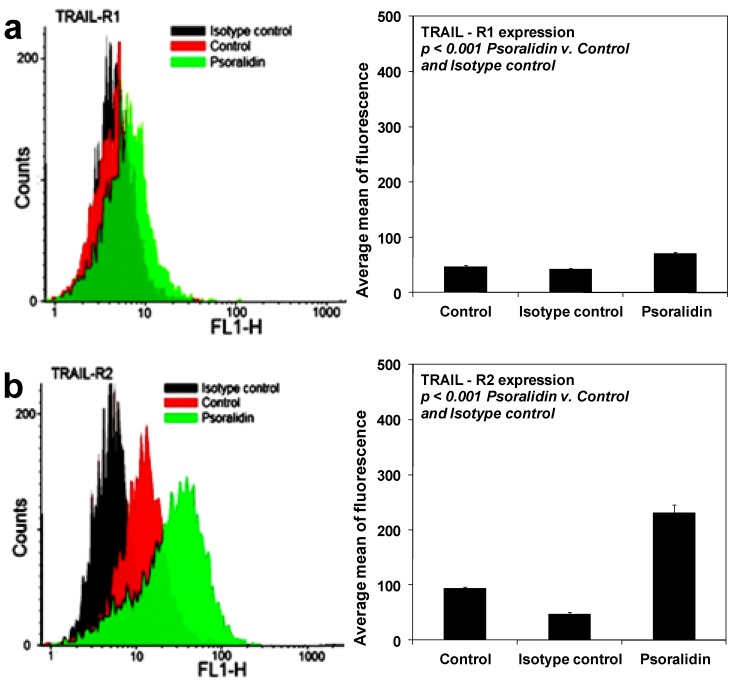
Effects of psoralidin on death receptor expression in HeLa cancer cells. Cells were incubated for 48 h with psoralidin at the concentration of 50 μM. The surface expression of (**a**) TRAIL-R1 and (**b**) TRAIL-R2 on cancer cell was measured by flow cytometry. The values represent mean ± SD of three independent experiments performed in duplicate (n = 3).

We analyzed the expression of TRAIL-R1 and TRAIL-R2 in HeLa cells after a 48-hour treatment with 50 μM psoralidin by flow cytometry (Figure 6). Psoralidin significantly increased TRAIL-R2 and slightly TRAIL-R1 expression in HeLa cell surface. The compound sensitized cancer cells to TRAIL through the extrinsic (receptor) apoptotic pathway via up-regulation of TRAIL-R2. 

Our results corroborate those of previous studies showing that treatment of DU-145 and PC-3 prostate cancer cells with psoralidin markedly increase the expression of death receptor TRAIL-R1 and TRAIL-R2 [11]. Esculetin augmented TRAIL-induced apoptosis in SAS oral cancer cells by up-regulation of TRAIL-R2 [42].

#### 2.4.2. Effects of TRAIL and Psoralidin on Mitochondrial Membrane Potential (ΔΨm) in HeLa Cancer Cells

Mitochondrial membrane depolarization is one of the first intracellular changes following the onset of apoptosis [16,17]. We evaluated whether psoralidin sensitizes cancer cells to TRAIL-induced mitochondrial dysfunction.

**Figure 7:**
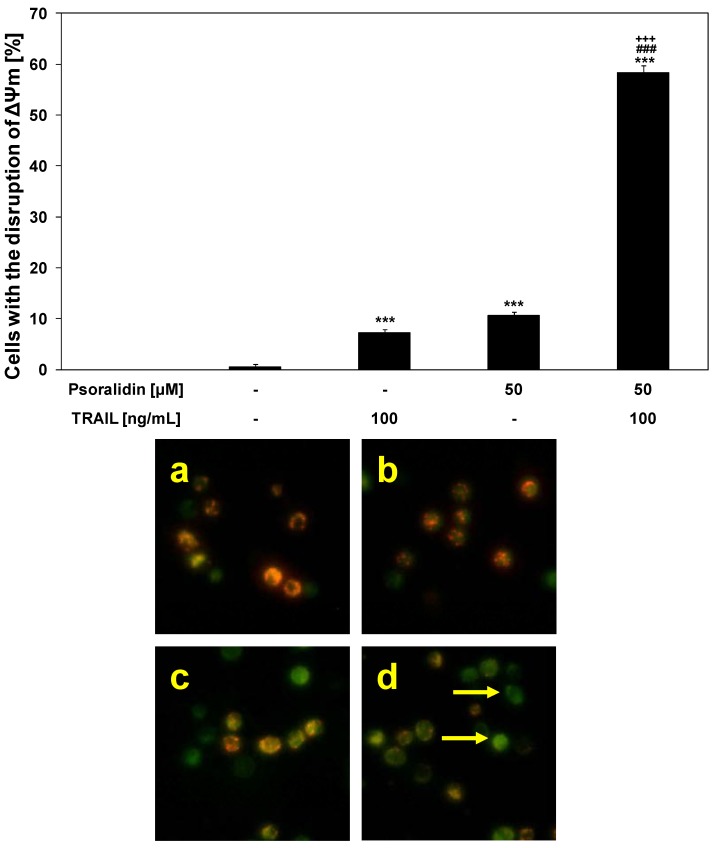
Effects of TRAIL in combination with psoralidin on the mitochondrial membrane potential (ΔΨm) in HeLa cancer cells. Cells were incubated for 48 h with TRAIL at the concentration of 100 ng/mL and/or psoralidin at the concentration of 50 μM. The values represent the mean ± SD of three independent experiments performed in duplicate n = 3 (***p < 0.001 compared with control, ^###^*p* < 0.001 compared with Psoralidin and ^+++^*p* < 0.001 compared with TRAIL). TRAIL in combination with psoralidin induced loss of ΔΨm in HeLa cells. Disruption of ΔΨm in cancer cells was assessed by fluorescent microscopic analysis of DePsipher staining: (**a**) control cells; (**b**) cells incubated with TRAIL; (**c**) cells incubated with psoralidin; (**d**) cells incubated with TRAIL and psoralidin. Red fluorescence is emitted from the red aggregates of DePsipher, which are formed within mitochondria in healthy cells. Green fluorescence reveals the monomeric form of the DePsipher molecule, which appears in the cytosol after mitochondrial membrane depolarisation (indicated by arrows).

The treatment of HeLa cells with 100 ng/mL TRAIL or 50 μM psoralidin alone caused little effect on ΔΨm (7.25 ± 0.71% and 10.63 ± 0.74%, respectively). The combination of TRAIL with psoralidin enhanced ΔΨm loss in a large percentage of cancer cells (58.38 ± 1.41%) and induced a significant disruption of the ΔΨm (Figure 7). These results demonstrated the engagement also of the intrinsic (mitochondrial) apoptotic pathway in HeLa cells co-treated with TRAIL and psoralidin.

Srinivasan *et al.* described a significant increase in the expression of cytochrome c in DU-145 and PC-3 cells after exposure of psoralidin, indicating the involvement of mitochondrial membrane potential. Additionally, psoralidin treatment causes a decrease of total Bid, that suggest the crosstalk between both apoptotic pathways, the extrinsic (receptor) and intrinsic (mitochondrial). Psoralidin alters in cancer cells, both receptor-mediated and mitochondrial, signals lead to apoptosis [11]. In contrast, Kok *et al.* showed that esculetin does not influence the mitochondrial-dependent pathway in SAS cells [42].

## 3. Experimental

### 3.1. Coumarin

Psoralidin was obtained from Alexis Biochemicals (San Diego, CA, USA). The compound was dissolved in DMSO (50 mM) to a final concentration of 0.1% (*v/v*) in the culture media. 

### 3.2. TRAIL

Soluble recombinant human TRAIL (rhsTRAIL) was purchased from PeproTech Inc. (Rocky Hill, NJ, USA).

### 3.3. Cancer Cell Culture

The experiments were performed on a HeLa human cervical cancer cell line (DSMZ (Deutsche Sammlung von Mikroorganismen und Zellkulturen) GmbH-German Collection of Microorganism and Cell Cultures, Braunschweig, Germany). The HeLa cells were grown in monolayer cultures in RPMI 1640 containing 10% fetal bovine serum (FBS) with 4 mM L-glutamine, 100 U/mL penicillin and 100 μg/mL streptomycin. The cancer cells were grown at 37 °C in atmosphere with 5% CO_2_ [34,35,36]. All reagents for cell culture were obtained from PAA Laboratories (Pasching, Austria).

### 3.4. Cytotoxicity Assay

The cytotoxicity was measured by the 3-(4,5-dimethylthiazol-2-yl)-2,5-diphenyltetrazolium bromide (MTT) assay as described previously [48,49]. The HeLa cells (2.5 × 105/mL) were seeded 24 h before the experiments in a 96-well plate. Psoralidin (20–50 μM) and/or TRAIL (50–200 ng/mL) were added to the cells, and 48 h later the medium was removed and 20 μL MTT solutions prepared at 5 mg/mL (Sigma Chemical Company, St. Louis, MO, USA) were added to each well for 4 h. The resulting crystals were dissolved in DMSO. Controls included native cells and medium alone. The spectrophotometric absorbance of each well was measured using a microplate reader (ELx 800, Bio-Tek Instruments Inc., Winooski, VT, USA) at 550 nm. The percent cytotoxicity was calculated by the formula: percent cytotoxicity (cell death) = [1 − (absorbance of experimental wells/absorbance of control wells)] × 100%.

### 3.5. Lactate Dehydrogenase Release Assay

Lactate dehydrogenase (LDH) is a stable cytosolic enzyme that is released upon membrane damage in necrotic cells. LDH activity was measured using a commercial cytotoxicity assay kit (Roche Diagnostics GmbH, Mannheim, Germany), in which LDH released in culture supernatants is measured with a coupled enzymatic assay, resulting in conversion of a tetrazolium salt into red formazan product. The HeLa cells were treated with various concentrations of psoralidin (20–50 μM) alone and in combination with TRAIL (50–200 ng/mL) for the indicated period of time. The sample solution (supernatant) was removed and LDH released from cells was measured in culture medium. The maximal release was obtained after treating control cells with 1% Triton X-100 (Sigma Chemical Company) for 10 min at room temperature [39,40,50,51]. The necrotic percentage was expressed using the formula: (sample value/maximal release) × 100%.

### 3.6. Determination of Apoptotic Cell Death by Fluorescence Microscopy with Annexin V-FITC Staining

Apoptotic cells were quantified by the fluorescence microscopy method using the Apoptotic & Necrotic & Healthy Cells Quantification Kit from Biotium, Inc. (Hayward, CA, USA) according to the manufacturer’s instruction [17,27,34,36]. The HeLa cells (2.5 × 10^5^/mL) were seeded 24 h before the experiments in a 24-well plate. Psoralidin (20–50 μM) and/or TRAIL (50–200 ng/mL) were added to the cancer cells, and 48 h later the cells were washed with PBS and detached from cell culture wells by trypsin. Next, the HeLa cells were centrifuged to discard supernatant, washed with PBS and resuspended in Binding Buffer (100 μL/sample). To each tube were added: 5 μL of Annexin V-FITC, 5 μL of Ethidium Homodimer III and 5 μL of Hoechst 33342 solutions. The samples were incubated at room temperature for 15 min in the dark. After staining the cancer cells were washed with Binding Buffer and placed on a glass slide and covered with a glass coverslip. The total number of 200 cells per sample was taken for analysis. The stained cells were observed under a fluorescence inverted microscope IX51 (Olympus, Tokyo, Japan) using filter set for FITC, TRITC and DAPI. The healthy cells (stained with Hoechst 33342) emitted blue fluorescence, apoptotic cells (stained with Annexin V-FITC and Hoechst 33342) emitted green and blue fluorescence and necrotic cells (stained with Ethidium Homodimer III and Hoechst 33342) emitted red and blue fluorescence. Cancer cells stained with triple colours blue, red and green, were dead cells progressing from apoptotic cell population. The cells were counted each time from the representative area containing 100 cells and apoptotic cells were expressed as percentage of total cells.

### 3.7. Flow Cytometric Analysis of Death Receptor Expression on the Cancer Cell Surface

The cell surface expression of death receptors TRAIL-R1 and TRAIL-R2 was determined by flow cytometry (LSR II, Becton Dickinson Immunocytometry Systems, San Jose, CA, USA). HeLa cells (2.5 × 10^5^/mL) were seeded in 24-well plates for 24 h and exposed to psoralidin (50 μM) for 48 h. Cells were then harvested using solution of trypsin and ethylenediaminetetraacetic acid (EDTA), washed twice in PBS and resuspended in PBS containing 0.5% bovine serum albumin (BSA). HeLa cells were incubated with 10 μL phycoerythrin-conjugated anti-TRAIL-R1 or anti-TRAIL-R2 monoclonal antibody (R&D Systems) at 4 °C for 45 min. After staining, the cells were washed with PBS and analysed using flow cytometry [26,47]. The control sample consisted of cells in a separate tube treated with phycoerythrin-labelled mouse IgG_1_ or mouse IgG_2B_ (R&D Systems).

### 3.8. Evaluation of Mitochondrial Membrane Potential by DePsipher

The DePsipher Kit (R&D Systems) was used to measure the mitochondrial membrane potential using fluorescence microscopy [17,24,47,51]. HeLa cells (2.5 × 10^5^/mL) were seeded in a 24-well plate 24 h prior to the experiments. TRAIL (100 ng/mL) and/or psoralidin (50 μM) were added, and 48 h later, the cells were washed with PBS and harvested by trypsinisation. The cells were incubated in the dark with DePsipher (5,5',6,6'-tetrachloro-1,1',3,3'-tetraethylbenzimidazolyl carbocyanin iodide) solution at a concentration of 5 μg/mL for 30 min at 37 °C, washed with reaction buffer with stabiliser, placed on a glass slide and covered with a glass cover slip. The stained cells were observed with a fluorescence inverted microscope using filter sets for FITC and TRITC. DePsipher undergoes potential-dependent accumulation in the mitochondria, which is indicated by a fluorescence emission shift from red (590 nm) to green (530 nm). 

### 3.9. Statistical Analysis

The results are expressed as means ± S.D. obtained from three separate experiments performed in duplicate or quadruplicate. Statistical significance was evaluated using ANOVA or t Student’s test. *P*-values < 0.05 were considered significant.

## 4. Conclusions

Psoralidin enhanced TRAIL-induced apoptosis in HeLa cells and overcame TRAIL-resistance by engaging both intrinsic and extrinsic apoptotic pathway via increase expression of TRAIL-R2 and induction of loss of ΔΨm. Together, our results suggest that psoralidin exerts their anticancer and chemopreventive effects through the modulation of TRAIL-mediated death in tumor cells.
